# Worry as a Cause of Indigestion

**Published:** 1898-09

**Authors:** 


### SELECTIONS.

### Worry as a Cause of Indigestion.

Worry is one of the most prolific causes of indigestion. Many patients present themselves to be relieved of dyspeptic troubles, to whom we give remedies which have served us well, and yet we effect no permanent cures. Why? Because the cause continues to operate.

Perhaps the patient sits down to a hurried, solitary meal, brooding over the snarl in his business while he munches. Or, perhaps, he is one of those unfortunate men whose families drag the household skeleton to the table with them.

In such cases the blood is not in the stomach where it is due to stimulate secretory activity, but in the head, thrashing over useless chaff. "Smart is the man who lets not his enemy worry him." Worry never did and never will accomplish anything save wear and tear. There is always a good side. Look for it and dwell on it. When you must take forethought, do not choose meal hours or the silent watches of the night.

Many a doctor goes to bed at night, and instead of closing the mind up promptly, he begins to review his day's work and to consider what he shall do on the morrow.

There is a time for everything. Be as orderly and systematic in the inner as well as the external life.

Give your patients a little philosophy along with your drugs. Good fellowship is the very best sauce for viands. It should be served daily in every home. The laugh and jest which drives away care insures digestion and prolongs life.—*Medical Brief.*

